# Annual Commencement of Buffalo University Medical College

**Published:** 1897-05

**Authors:** 


A Monthly Review of Medicine and Surgery.

EDITORS:

THOMAS LOTHROP, M. D. - - WM. WARREN POTTER, M. D.

All communications, whether of a literary or business nature, should be addressed to the managing editor: 284 FRANKLIN STREET, BUFFALO, N. Y.

VOL. XXXVI. MAY, 1897. No. 10.

### ANNUAL COMMENCEMENT OF BUFFALO UNIVERSITY MEDICAL COLLEGE.

### ALUMNI ASSOCIATION MEETING AND BANQUET.

THE fifty-first commencement exercises of the department of medicine of the University of Buffalo were held Tuesday, April 27, 1897. On the same day also the department of pharmacy held its tenth, and the department of dentistry its fifth commencement exercises. As usual on these occasions, the curators met in the forenoon to ratify the examinations and to legalise the recommendations of the faculty regarding candidates for degrees.

### ALUMNI ASSOCIATION.

The alumni association held its twenty-third annual meeting in Alumni hall of the college building in the afternoon of commencement day.

The president, Dr. P. W. Van Peyma, of Buffalo, called the meeting to order at 2 o'clock P. M. About fifty graduates were present from different sections of the country representing nearly every class that had been sent out since the school was organised in 1846. Dr. A. G. Ellenwood, of Attica, class of '48, was the first to respond when the roll was called by years. Dr. Thomas D. Strong, of Westfield, class of '61, first president of the alumni association, was in attendance, besides many others who graduated in the earlier years. Then came the election of officers for the ensuing year, with the following result: President, Dr. D. A. Currie, of Englewood, N. J.; first vice-president, Dr. J. J. Lusk, of Warsaw; second vice-president, Dr. W. W. Potter, of Buffalo; third vice-president, Dr. M. A. Veeder, of Lyons; fourth vice-president, Dr. J. W. Putnam, of Buffalo; fifth vice-president, Dr.

W. R. Campbell, of Niagara Falls. Trustees: Drs. Joseph Fowler, Buffalo; Julius Wenz, Lancaster; H. P. Trull, Williamsville; C. A. Wall, Buffalo, and W. C. Phelps, Buffalo. Executive committee: Drs. Albert T. Lytle, chairman; H. G. Matzinger, secretary, and G. W. Wende, Buffalo, and the president of the association and secretary of the faculty *ex-officio*.

After the completion of the business session the following scientific program was carried out: Dr. P. W. Van Peyma, the retiring president, delivered the annual address. He was followed by Dr. Alfred Stengel, of the Pepper laboratory of the University of Pennsylvania, who read a paper on The anatomy and pathology of the red blood corpuscle. Drs. H. U. Williams, H. G. Matzinger and A. E. Woehner joined in the discussion of Dr. Stengel's paper. Other papers which were read were: Treatment of neurasthenia, by Dr. James W. Putnam, of Buffalo, which was discussed by Drs. J. G. Van Pelt, W. M. Baker and Bina P. Vandenbergh; Hypertrophy of the prostate, by Dr. William C. Phelps, of Buffalo, discussed by Drs. A. G. Ellinwood, J. G. Kelly and W. R. Palmer. Following the papers Dr. Herbert M. Hill and Dr. H. U. Williams, of Buffalo, gave a microscope lantern exhibit, projecting a number of interesting specimens.

### EXERCISES AT MUSIC HALL.

The graduating exercises were held at Music hall at 8 o'clock in the evening, which was crowded long before the hour with an interested and fashionable audience. The classes of the several departments, in cap and gown (except the pharmacists), marched down the aisle promptly at the appointed hour to strains of inspiring music and greeted by the enthusiastic plaudits of 2,000 spectators.

The chaplain, Rev. Walter North, D. D., read from Ecclesiasticus, the 38th chapter, beginning: "Honor a physician with the honor due unto him, for the uses which ye may have of him; for the Lord hath created him." After prayer and another interlude Dr. John Parmenter, secretary of the medical department, offered the names of the candidates for the degree of doctor of medicine, as follows:

Ayers, Lemuel DeLos, Tonawanda, Pa.; Allen, Geo. S., Farmington, N. Y.; Bauer, Eugene E., Owego, N. Y.; Bacon, Chas. Bowman, New Lebanon, N. Y.; Baker, Amos Trevallee, Buffalo, N. Y.; Busch, Frederick C., B. S., Buffalo, N. Y.; Bair, Edmund A., Emporium, Pa.; Barnsdall, J. Thornton, Buffalo, N. Y.; Burns, Irving W., M. D., North Tonawanda, N. Y.; Burt, Fred C., Belfast, N. Y.; Barrett, Frederick J., Buffalo, N. Y.; Comfort,

Clifford V., Ph. B., Rochester, N. Y.; Culver, Clifton W., Buffalo, N. Y.; Craig, Charles Smith, Hilton, N. Y.; Cummings, Carro Julia, B. Ph., Buffalo, N. Y.; Clarke, Clayton Reynolds, Olean, N. Y.; Cowper, Harold William, Buffalo, N. Y.; Crittenden, Frederick G., Phelps, N. Y.; Croff, J. Brainard, Batavia, N. Y.; Dodge, W. Lee, West Westfield, N. Y.; DeGroat, Herman K., A. M., Buffalo, N. Y.; Evans, W. I. C., Machias, N. Y.; Eddy, Loren L., Olean, N. Y.; Elliott, John George, Gasport, N. Y.; Fronczak, Francis E., A. M., L. L. P., Buffalo, N. Y.; Garratt, John Milton, Buffalo, N. Y.; Goff, A. P., Cameron Mills, N. Y.; Hummel, Edward E., Williamsville, N. Y.; Hays, Mary J., Kushequa, Pa.; Hunt, Fred C., Sinclairville, N. Y.; Hendee, Lawrence, Buffalo, N. Y.; Halleck, Margaret S., A. B., Buffalo, N. Y.; Hoke, William A., Morris, N. Y.; Kiepe, Edward John, Ph. G., Buffalo, N. Y.; Keeler, J. Grant, Rochester, N. Y.; Kerr, A. T., Jr., B. S., Buffalo, N. Y.; Leslie, Wm. G., Rochester, N. Y.; Loughlen, John J., Olean, N. Y.; Leary, James H., Henrietta, N. Y.; Lock, Frank E., Buffalo, N. Y.; Loop, Ross G., Elmira, N. Y.; Love, Frank W., B. S., Buffalo, N. Y.; LaWall, Elbert W., Corning, N. Y.; Lansing, Levinus A., Buffalo, N. Y.; McIntosh, George, Seaforth, N. Y.; MacKenzie, Simon S., Caledonia, N. Y.; MacPherson, Marjory J., N. Tonawanda, N. Y.; McWayne, E. P., Sackett's Harbor, N. Y.; Mastin, Charlotte E., Wellsboro, Pa.; Means, George Sherwood, Geneva, N. Y.; Mendlein, Fred A., Dansville, N. Y.; Minard, George H., Buffalo, N. Y.; O'Leary, James F., A. B., Hartford, Conn.; Palmer, Wm. E., York, N. Y.; Palmer, Wm. A., Ph. G., Elmira, N. Y.; Potter, Will H., Springville, N. Y.; Phillips, William L., Buffalo, N. Y.; Parker, Burt T., Buffalo, N. Y.; Potter, Alice May, Ithaca, N. Y.; Remington, Alvah Childs, West Shelby, N. Y.; Rosengren, Charles J., Buffalo, N. Y.; Ross, Fred Ernest, Erie, Pa.; Ryle, John J., A. B., Stamford, Conn.; Soch, Albert F., Smith Mills, N. Y.; Turk, George R., Clarence, N. Y.; Trotter, Richard, Ph. G., Buffalo, N. Y.; Wickens, Vallance A., A. B., Rochester, N. Y.; Witt, Ervin W., Watertown, N. Y.; Walker, Townsend, Bath, N. Y.; Williams, C. S., Cayutaville, N. Y.; Woolston, Clayton S., Gaines, N. Y.; Westlake, Carolyno Louise, Le Roy, N. Y.

The Hippocratic oath was administered by Dr. Matthew D. Mann, the dean of the medical faculty. Then the candidates filed upon the stage and the chancellor, Hon. James O. Putnam, conferred the degrees in these words :

"By virtue of the authority in me vested as chancellor of the University, I confer upon each of you, individually and severally, the degree of Doctor of Medicine, in token of which I give you this diploma."

The custom of the medical department is to place the names of all those who have made an average mark of 90 per cent. or over during their course on the honor roll. The following names were so placed : Amos Trevallee Baker, A. P. Goff, Fred Ernest Ross, Frank E. Lock, Margaret S. Halleck, A. B. ; Carro Julia Cummings, B. Ph. ; Lawrence Hendee, Eugene E. Bauer, Albert F. Soch, Frank W. Love, B. S.

The following names, with the titles of their graduating theses, were announced as deserving honorable mention : Cultivation of the gonococcus, by Frederick C. Busch ; Membranous colites, by Miss Carro Julia Cummings ; Plica polonica, by Francis E. Fronczak, A. M., LL. P. ; Multiple serositis, by Miss Margaret S. Halleck ; Salivary digestion, by Edward John Kiepe, Ph. G. ; The fate of animal sutures in the tissues, by Lawrence Hendee ; The common reagents for the detection of albuminuria, by John M. Garratt ; A plea for the Greek ideal of beauty, by Alice May Potter ; Paranoia, by Ross G. Loop ; The comparative anatomy of the cecum, by Abram T. Kerr ; A microscopic study of the early development of the embryo, by Frank W. Love.

Dr. John R. Gray, secretary of the department of pharmacy, presented the candidates for the degree of Ph. G., which was conferred by Chancellor Putnam upon a class numbering forty-six, including four who received the degree of Master in Pharmacy and two who met all requirements excepting that of age, and who merely received certificates of examination.

Dr. A. P. Southwick presented the names of sixty-eight dentists upon whom the degree of D. D. S. was conferred by Chancellor Putnam.

After a musical selection Prof. Joseph P. Remington, Ph. M., F. C. S., dean of the Philadelphia College of Pharmacy, addressed the graduating classes. In a scholarly effort Dr. Remington traced the growth of modern methods of instruction in pharmacy, which in this country had its origin at Philadelphia, in 1821, as the result of a chance meeting and conversation between Peter Lehman and Henry Troth, two esteemed druggists in that city. The speaker gracefully referred to the other branches of curative science and art represented in the classes before him, giving each friendly words of wise suggestion and advice. A benediction by the chaplain closed the formal commencement exercises of 1897.

### ALUMNI BANQUET.

The annual banquet of the alumni association was given at the Ellicott club. A few minutes after 11 o'clock orchestral strains announced everything in readiness, when the faculty and alumni marched into the spacious dining-room of the Ellicott club—the men from one side and the women from the other—taking seats at small tables in groups according to their own assortment. N. W. Wilson, Jacob Otto, W. C. Gerber, George Staniland, Harvey Albertson and Guy Boughton, members of the class of '98, acted as ushers. At a large round table, handsomely decorated, sat the president of the alumni, Dr. P. W. Van Peyma, Rev. Walter North, D. D., Dr. M. D. Mann, Prof. Remington, of Philadelphia, Dr. Thomas D. Strong, of Westfield, Dr. Roswell Park, Dr. H. M. Hill, Dr. Charles G. Stockton, Dr. John Parmenter, Dr. Woods Hutchinson and Dr. William Warren Potter. After an excellent service, the following toasts were announced by Dr. Van Peyma, who announced each speaker in bright, witty and appropriate sentences : State medical examinations for license to practise, Dr. William Warren Potter, of Buffalo ; The patient, Dr. Charles H. Richmond, of Livonia Station, N. Y. ; 1897, Dr. Frederick C. Busch, of Buffalo ; Me judice, Dr. Maud J. Frye, of Buffalo ; Newspaper medicine, Dr. Woods Hutchinson, of Buffalo.

When the president in the small hours announced the meeting as over, it was the general opinion expressed by many who have frequently attended these annual gatherings that 1897 bore off the palm. For the successful management of the alumni meeting, afternoon and evening sessions, the executive committee, under the able chairmanship of Dr. Albert T. Lytle, is entitled to all praise.

